# Cardiorespiratory Arrest in the Context of Graves' Disease: A Rare Presentation

**DOI:** 10.7759/cureus.118542

**Published:** 2026-10-25

**Authors:** Mariana Marques, Joana Couto, Madalena Martins Vale, Pedro Pinto, Paula Felgueiras

**Affiliations:** 1 Internal Medicine, Hospital de Vila Franca de Xira, Vila Franca de Xira, PRT; 2 Internal Medicine, Unidade Local de Saúde do Alto Minho, Viana do Castelo, PRT

**Keywords:** graves' disease, implantable cardioverter-defibrillator, sudden cardiac arrest, thyroid storm, ventricular fibrillation

## Abstract

Thyroid storm is the most severe and life-threatening expression of thyrotoxicosis, and its diagnosis remains clinical. Although sinus tachycardia and atrial fibrillation dominate its cardiovascular manifestations, malignant ventricular arrhythmias are rare, and cardiorespiratory arrest as the presenting manifestation of Graves' disease has been reported only in a limited number of cases.

We report the case of a 44-year-old man with a one-week history of symptoms consistent with hyperthyroidism who suffered a witnessed out-of-hospital cardiorespiratory arrest with ventricular fibrillation as the initial rhythm. Return of spontaneous circulation was achieved after three defibrillations, and urgent coronary angiography showed no obstructive coronary artery disease. Laboratory evaluation revealed severe thyrotoxicosis with positive thyroid-stimulating hormone (TSH)-receptor antibodies, and thyroid ultrasound findings supported the diagnosis of Graves' disease. Thyroid storm was suspected and treated empirically, but the diagnosis could not be confirmed: the Burch-Wartofsky score reached the diagnostic threshold only when post-arrest coma was included, and the Japan Thyroid Association criteria were not met. Cardiac magnetic resonance imaging and an ajmaline provocation test did not identify structural heart disease, myocarditis, or a Brugada pattern. Thyroid hormone excess was therefore considered the most likely precipitant of the arrhythmia, although causality cannot be proven. The patient was maintained on antithyroid therapy, and a subcutaneous implantable cardioverter-defibrillator was implanted for secondary prevention following an individualized risk-benefit assessment.

This case highlights the importance of considering thyrotoxicosis in the etiological workup of unexplained cardiac arrest. When a clearly established and reversible cause of the arrhythmia cannot be confirmed, management, including the decision on defibrillator implantation, should be based on an individualized risk-benefit assessment.

## Introduction

Graves' disease is the most common cause of hyperthyroidism in iodine-sufficient regions and results from thyrotropin receptor-stimulating autoantibodies that drive unregulated thyroid hormone synthesis and secretion [1]. While most patients present with the typical clinical features of thyrotoxicosis (weight loss, palpitations, tremor, and heat intolerance), a small proportion progress to thyroid storm, the most extreme and life-threatening expression of the disease. Thyroid storm is rare, with an estimated incidence of 0.57-0.76 cases per 100,000 persons per year, and carries a mortality of 10-30% even with modern intensive care [2,3].

The diagnosis of thyroid storm remains clinical, supported by semi-quantitative tools such as the Burch-Wartofsky Point Scale (BWPS) and the Japan Thyroid Association criteria, which weigh thermoregulatory, cardiovascular, gastrointestinal-hepatic, and central nervous system dysfunction [2,4]. Cardiovascular involvement is the dominant driver of mortality: thyroid hormone excess increases heart rate, contractility, and myocardial oxygen demand, shortens myocardial refractory periods, and heightens sensitivity to catecholamines, predisposing to tachyarrhythmias, most commonly sinus tachycardia and atrial fibrillation, as well as high-output heart failure [5].

Malignant ventricular arrhythmias are, by contrast, a rare manifestation of thyrotoxicosis, and cardiorespiratory arrest as the presenting manifestation of Graves' disease has been reported only in a limited number of cases [6-9]. This presentation poses a dual challenge: recognizing thyrotoxicosis as a potentially reversible cause of the arrest, and instituting specific therapy in a patient whose hemodynamic condition constrains conventional options. We report a case of previously undiagnosed Graves' disease presenting with cardiorespiratory arrest in ventricular fibrillation and discuss the difficulty of attributing cardiac arrest to a potentially reversible endocrine cause and the subsequent management of arrhythmic risk, including the decision regarding defibrillator implantation.

## Case presentation

A 44-year-old man, fully independent, an active smoker with a 20 pack-year history, with no other relevant past medical history and on no regular medication, presented with a one-week history of palpitations, profuse sweating, and elevated blood pressure. There were no weight loss, heat intolerance, tremor, goiter, known thyroid disease, or family history of sudden cardiac death and no gastrointestinal symptoms or clinical signs of heart failure. An acute alteration of consciousness at home was witnessed by his wife; on arrival of the emergency medical team, he was found in cardiorespiratory arrest with ventricular fibrillation as the initial rhythm. Return of spontaneous circulation (ROSC) was achieved after a total of three defibrillation attempts, amiodarone 300 mg, and epinephrine 1 mg, and the patient was intubated in the prehospital setting.

The post-ROSC electrocardiogram showed sinus tachycardia (116 bpm) with ST-segment elevation in the septal and lateral leads, a QTc of 440 ms, and no other conduction or repolarization abnormalities. The patient was hypertensive (184/111 mmHg); his temperature was 37.4°C, and his arterial oxygen saturation was 98% under invasive mechanical ventilation. He was transferred to a hospital with interventional cardiology capability, where urgent coronary angiography revealed a left-dominant circulation with no evidence of obstructive coronary artery disease. Arterial blood gas analysis showed a lactate of 4.46 mmol/L and mild type II respiratory failure without acidemia or electrolyte disturbances. Laboratory workup revealed hemoglobin 15.9 g/dL, no leukocytosis, platelets 296,000/µL, creatinine 1.0 mg/dL, urea 38 mg/dL, sodium 137 mmol/L, potassium 5.0 mmol/L, calcium 8.9 mg/dL, magnesium 2.1 mg/dL, elevated transaminases (aspartate aminotransferase (AST) 387 U/L, alanine aminotransferase (ALT) 278 U/L) with normal bilirubin, alkaline phosphatase 217 U/L, creatine kinase 276 U/L, elevated cardiac troponin (2.26 ng/mL), brain natriuretic peptide (BNP) 169 pg/mL, and C-reactive protein 3.21 mg/dL. Blood alcohol and urine drug screening were negative, and urinalysis was unremarkable. Infection, recent medication changes, and prior exposure to iodinated contrast were excluded as precipitants. Thyroid function testing showed suppressed thyrotropin (thyroid-stimulating hormone (TSH) <0.008 µIU/mL) and elevated free thyroxine (fT4 3.31 ng/dL). The admission laboratory findings are summarized in Table 1.

**Table 1:** Laboratory findings at admission AST: aspartate aminotransferase; ALT: alanine aminotransferase; BNP: brain natriuretic peptide; TSH: thyroid-stimulating hormone; TRAb: TSH-receptor antibody

Parameter	Value	Reference range
Hemoglobin (g/dL)	15.9	13.0-17.5
Platelets (/µL)	296,000	150,000-400,000
Creatinine (mg/dL)	1.0	0.7-1.2
Urea (mg/dL)	38	10-50
Sodium (mmol/L)	137	136-145
Potassium (mmol/L)	5.0	3.5-5.1
Calcium (mg/dL)	8.9	8.4-10.2
Magnesium (mg/dL)	2.1	1.8-2.6
AST (U/L)	387	<34
ALT (U/L)	278	<49
Alkaline phosphatase (U/L)	217	40-150
Creatine kinase (U/L)	276	<190
Cardiac troponin (ng/mL)	2.26	<0.03
BNP (pg/mL)	169	<100
C-reactive protein (mg/dL)	3.21	<0.5
Lactate (mmol/L)	4.46	0.5-2.2
TSH (µIU/mL)	<0.008	0.4-4.0
Free T4 (ng/dL)	3.31	0.8-1.8
Free T3 (pg/mL)	>20	2.0-4.4
TRAb (IU/L)	11	<1.8

Cranial computed tomography (CT) showed no ischemic or hemorrhagic lesions (Figure 1A). Thoraco-abdominopelvic CT revealed mild basal atelectasis and centrilobular emphysema (Figure 1B-1C), with a normal liver, no biliary dilatation, and unremarkable kidneys and adrenal glands (Figure 1D-1E).

**Figure 1:**
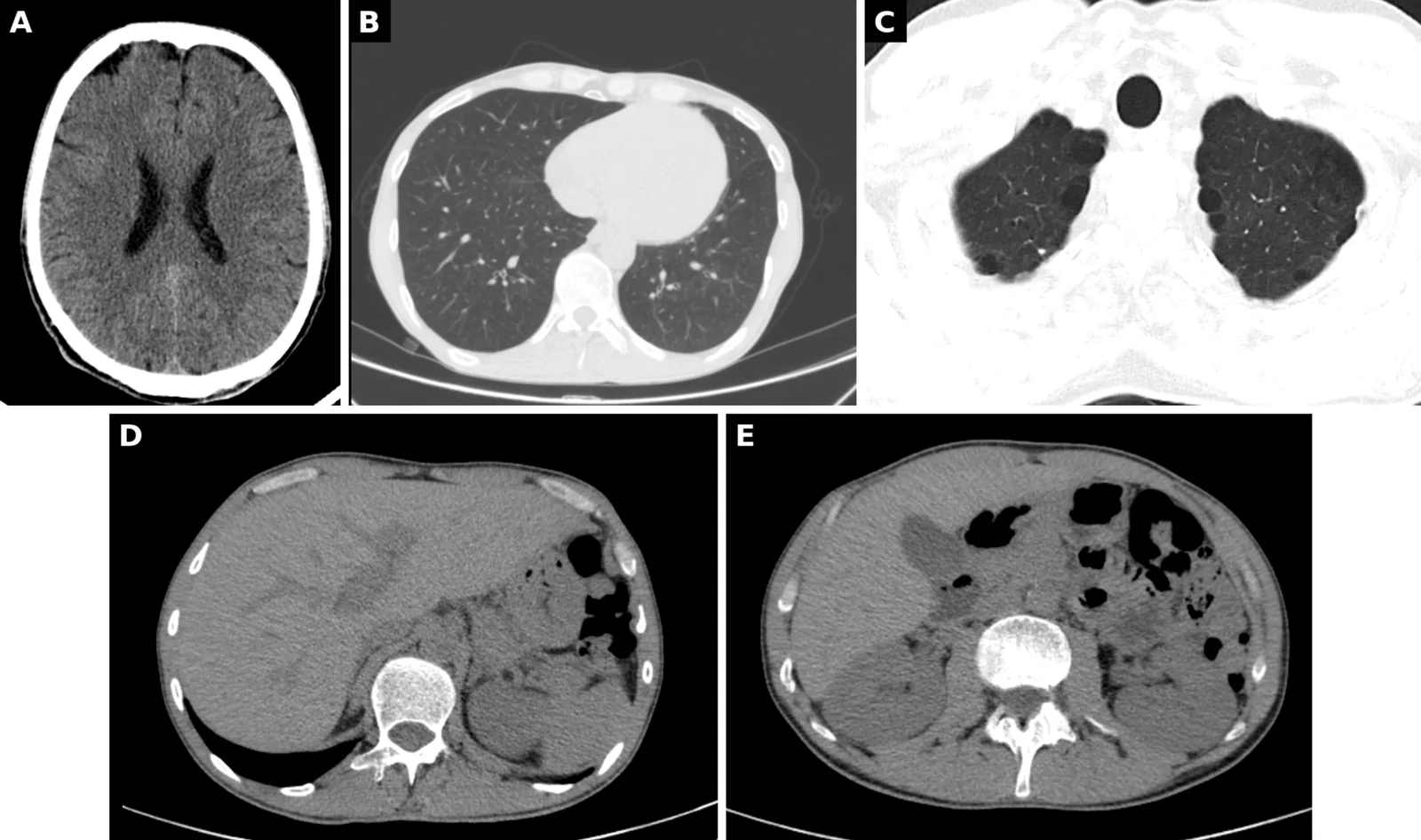
Cranial and thoraco-abdominopelvic CT (A) Axial non-contrast cranial CT showing no ischemic or hemorrhagic lesions. (B) Axial chest CT (lung window) at the lung bases showing mild basal atelectasis. (C) Axial chest CT (lung window) at the lung apices showing emphysematous changes. (D) Axial abdominal CT showing a normal liver without biliary dilatation. (E) Axial abdominal CT showing unremarkable kidneys. CT: computed tomography

Transthoracic echocardiography demonstrated non-dilated cardiac chambers, no valvular or segmental wall motion abnormalities, good biventricular systolic function, and no pericardial effusion (Figure 2).

**Figure 2:**
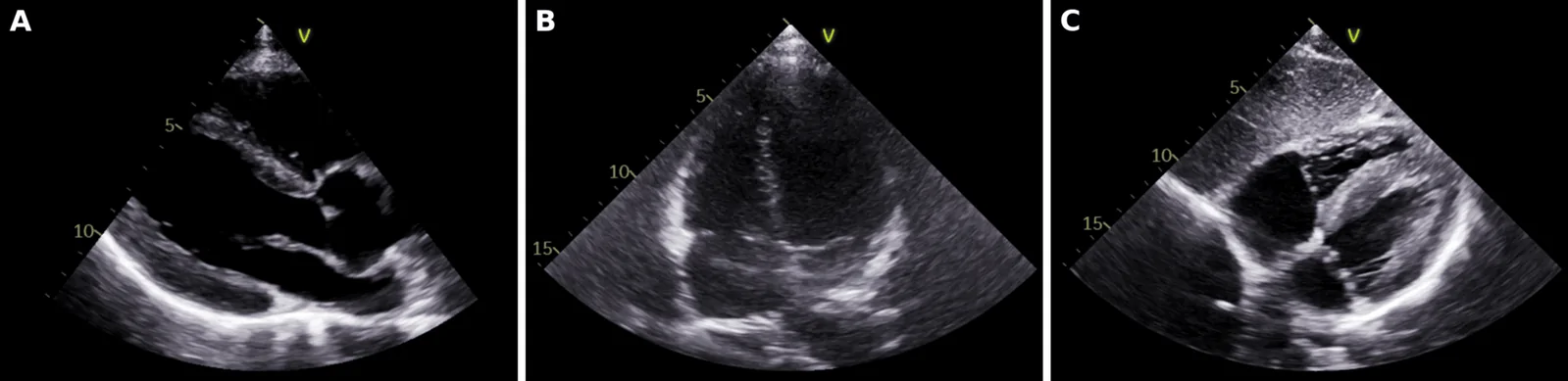
Transthoracic echocardiography (A) Parasternal long-axis view, (B) apical four-chamber view, and (C) subcostal four-chamber view showing non-dilated cardiac chambers, preserved biventricular systolic function, no valvular or segmental wall motion abnormalities, and no pericardial effusion.

Thyroid ultrasound revealed a diffusely enlarged gland (right lobe 25×27×60 mm; left lobe 19×19×56 mm) with lobulated contours and a diffusely heterogeneous echotexture, with increased vascularity on Doppler study and no nodular lesions or laterocervical adenomegaly (Figure 3), findings consistent with diffuse autoimmune thyroid disease.

**Figure 3:**
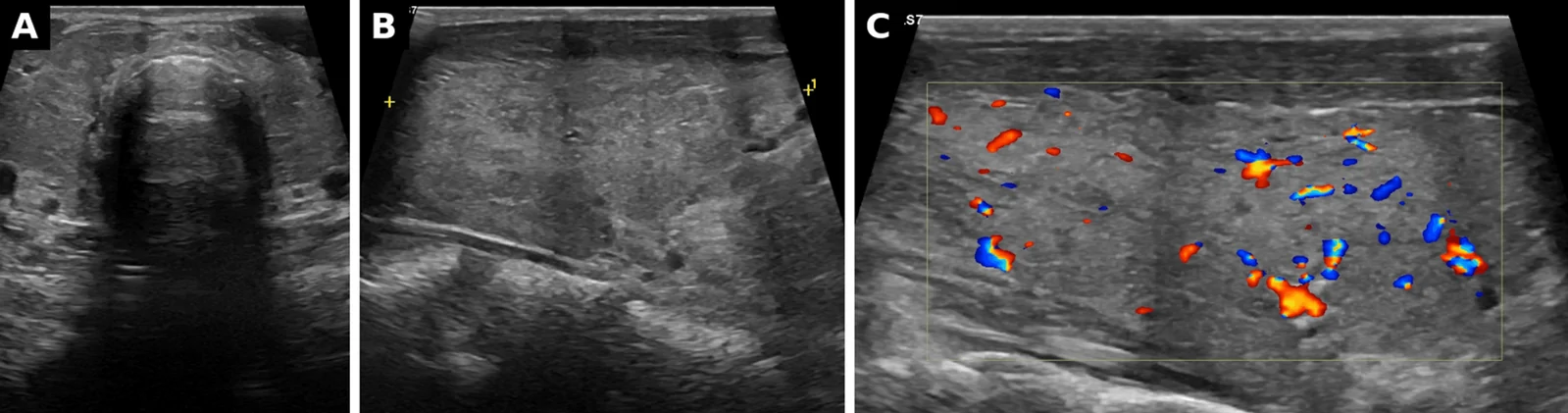
Thyroid ultrasound (A) Transverse grayscale view showing a diffusely enlarged thyroid gland with lobulated contours and heterogeneous echotexture, without nodular lesions. (B) Longitudinal grayscale view with measurement of the lobe length. (C) Longitudinal color Doppler view showing diffusely increased parenchymal vascularity.

Post-cardiac arrest status in the probable context of hyperthyroidism was assumed, and therapy was immediately initiated with propranolol (80 mg every eight hours), propylthiouracil (1 g loading dose followed by 200 mg every four hours), and hydrocortisone (300 mg bolus followed by 100 mg every eight hours) for suspected thyroid storm. Subsequent results showed markedly elevated free triiodothyronine (fT3 >20 pg/mL) and positive TSH-receptor antibodies (TRAb 11 IU/L), which, together with the ultrasound findings, supported the diagnosis of Graves' disease. The BWPS score was 45 points (temperature 37.2-37.7°C, 5 points; heart rate 110-119 bpm, 10 points; severe central nervous system dysfunction, 30 points), reaching the threshold suggestive of thyroid storm only because of the post-arrest neurological component; excluding this component, the score was 15 points (storm unlikely). No fever or independent pre-arrest central nervous system dysfunction was documented, and the Japan Thyroid Association criteria for definite (TS1) or suspected (TS2) thyroid storm were not met (no fever, heart rate below 130 bpm, no congestive heart failure, and no gastrointestinal or hepatic manifestations) [2]. The case was therefore classified as severe thyrotoxicosis with cardiac arrest in which thyroid storm was suspected at presentation and treated empirically, but the diagnosis could not be confirmed. Cardiac magnetic resonance imaging, performed in the second week of admission, showed no necrosis, fibrosis, or myocarditis. Cardiorespiratory arrest in the context of Graves' disease with severe thyrotoxicosis (thyroid storm suspected at presentation but not confirmed) was therefore assumed as the working diagnosis, with thyroid hormone excess considered the most plausible, but unproven, precipitant. Therapy was subsequently transitioned to methimazole (10 mg every 12 hours), prednisolone (40 mg daily), and propranolol (40 mg every eight hours), and a subcutaneous implantable cardioverter-defibrillator (S-ICD) was implanted approximately two months after the index event for secondary prevention, as an individualized decision given the uncertain reversibility of the arrhythmogenic mechanism (see Discussion).

The patient was extubated at 48 hours without complications, with no neurological deficits, and completed an uneventful ventilatory wean with no need for supplemental oxygen. Free thyroid hormone levels progressively improved under the therapy instituted, and he was discharged on propranolol 40 mg every eight hours, methimazole 5 mg twice daily, and prednisolone 40 mg daily, subsequently tapered slowly; at the time of writing, he remains on a tapering schedule and is currently taking prednisolone 5 mg daily. Over the following months of outpatient follow-up, antithyroid and beta-blocker therapy were serially titrated. Thyrotropin remained suppressed until month 8, with fluctuating free hormone levels (free T3 above the reference range at months 1, 2, and 6 and free T4 below it at months 4 and 8; Table 2). TSH-receptor antibody titers, which were 11 IU/L at admission, peaked at 23 IU/L at month 1 (same assay as at admission) and then declined progressively to 4.73 IU/L at month 9. At approximately nine months after the index event, TSH became detectable for the first time (0.35 µIU/mL, just below the lower reference limit of 0.4 µIU/mL), with free T4 at 1.10 ng/dL and free T3 at 3.2 pg/mL. An electrophysiological study with ajmaline provocation, performed approximately nine months after the index event and after S-ICD implantation, when free thyroid hormone levels were within the reference range but TSH remained below it (0.35 µIU/mL), did not induce a Brugada pattern, making Brugada syndrome unlikely, although not excluded. On follow-up examination, visible proptosis without inflammatory signs was noted, consistent with Graves' orbitopathy, and no further arrhythmic events or device therapies were documented. The patient currently awaits total thyroidectomy as definitive treatment of Graves' disease.

**Table 2:** Evolution of thyroid function and TSH-receptor antibody titers under therapy TSH: thyroid-stimulating hormone; TRAb: TSH-receptor antibody

Time point	TSH (µIU/mL)	Free T3 (pg/mL)	Free T4 (ng/dL)	TRAb (IU/L)
Reference range	0.4-4.0	2.0-4.4	0.8-1.8	<1.8
Admission	<0.008	>20	3.31	11
Month 1	<0.01	7.79	1.51	23
Month 2	<0.01	8.44	1.71	-
Month 4	<0.01	3.55	0.68	19
Month 6	<0.01	5.04	1.25	14
Month 8	<0.01	2.95	0.68	7.7
Month 9	0.35	3.2	1.10	4.73

## Discussion

Thyroid storm represents the extreme end of the thyrotoxicosis spectrum, occurring in approximately 1% of patients with hyperthyroidism, and remains a clinical diagnosis: no laboratory threshold distinguishes it from uncomplicated thyrotoxicosis, and free hormone levels overlap substantially between the two states [2,4,10]. Its presentation may be dominated, or masked, by the precipitating event or by a single organ system, which frequently delays recognition [11]. The BWPS attributes points for thermoregulatory, cardiovascular, gastrointestinal-hepatic, and central nervous system dysfunction, with a score of 45 or greater highly suggestive of storm and 25-44 suggestive of impending storm [4,10]. In the present case, a temperature of 37.4°C (5 points), a heart rate of 116 bpm (10 points), and loss of consciousness (severe central nervous system dysfunction, 30 points) yield 45 points, exactly at the threshold, but the score falls to 15 points, a range in which storm is unlikely, when the post-arrest neurological component is excluded. Loss of consciousness in the setting of cardiac arrest is not specific for thyroid storm and may be entirely attributable to cerebral hypoperfusion; no fever and no independent pre-arrest central nervous system dysfunction were documented. The Japan Thyroid Association criteria for definite (TS1) or suspected (TS2) thyroid storm [2] were also not met: there was no fever, the heart rate was below 130 bpm, and there was no congestive heart failure or gastrointestinal or hepatic manifestation, so the only criterion possibly present was a central nervous system manifestation, itself confounded by the arrest. The diagnosis of thyroid storm is therefore not independently supported by other clinical features, and we classify this case as severe thyrotoxicosis with cardiac arrest in which thyroid storm was suspected at presentation and treated empirically, but the diagnosis could not be confirmed. The one-week prodrome of palpitations, diaphoresis, and hypertension is consistent with accelerating thyrotoxicosis. The elevated transaminases may reflect hypoxic hepatitis after the arrest, thyrotoxic hepatic dysfunction, or both; pre-arrest liver function tests were not available. Abdominal CT showed a normal liver without biliary dilatation (Figure 1D), viral serologies obtained at admission because of the transaminase elevation were negative, and transaminases decreased progressively under treatment, reaching normal values by month 4 (AST 52 U/L and ALT 54 U/L at month 3; AST 30 U/L and ALT 36 U/L at month 4), which is compatible with either mechanism.

The cardiovascular system bears the brunt of thyroid hormone excess: increased heart rate and contractility, reduced systemic vascular resistance, shortened atrial and ventricular refractory periods, and heightened sensitivity to catecholamines [5]. Sinus tachycardia and atrial fibrillation are the dominant arrhythmias, whereas ventricular fibrillation is a rare manifestation of hyperthyroidism. An arrhythmogenic potential of thyroid hormone excess is, however, suggested by epidemiological data: in a prospective population-based cohort, higher free thyroxine levels were independently associated with sudden cardiac death, even within the euthyroid range [12].

The ST-segment elevation with angiographically normal coronary arteries observed in our patient deserves comment. Severe coronary artery spasm has been associated with hyperthyroidism and may precipitate ischemia and malignant ventricular arrhythmias in thyrotoxic patients with normal coronary anatomy [13]. A vasospastic contribution to the arrest cannot be excluded: no provocative coronary testing was performed, and the absence of myocardial scarring on cardiac magnetic resonance imaging does not rule out transient ischemia due to coronary vasospasm, which therefore remains a potential contributing factor. Active smoking, present in our patient, is a possible additional contributor to vasospasm.

The etiological workup was extensive. Sudden cardiac arrest has been reported as the presentation of Brugada syndrome unmasked by thyroid storm [14], a differential of direct relevance here. In our patient, an ajmaline provocation test performed approximately nine months after the index event did not induce a Brugada pattern. At that time, free thyroid hormone levels were within the reference range, although TSH remained below it (0.35 µIU/mL), so euthyroidism had not been fully achieved. Performing the test outside the acute thyrotoxic state makes a false-negative result less likely, but a single negative provocation test does not exclude Brugada syndrome, which we therefore consider unlikely rather than excluded. The test was performed after S-ICD implantation, which had been decided at approximately two months because of the risk of recurrent ventricular fibrillation, so its result did not inform that decision. Coronary angiography and cardiac magnetic resonance imaging did not identify structural or inflammatory heart disease, and the electrophysiological study did not reveal a Brugada pattern, although primary electrical heart disease cannot be completely excluded. Thyroid hormone excess therefore remains the most plausible, although unproven, precipitant. Plausible mechanisms, namely, shortened refractory periods, heightened catecholamine sensitivity, and possible coronary vasospasm, are well described, but no specific biomarker or test can demonstrate that thyroid hormone excess was directly responsible for the ventricular fibrillation, and causality remains an inference by exclusion. Prior reports of ventricular fibrillation complicating Graves' disease in patients without structural heart disease, including a series of three men [15], are consistent with this interpretation, but they are very few and do not establish causality.

Management followed the established pillars of thyroid storm therapy, applied on clinical suspicion: inhibition of hormone synthesis with a thionamide, blockade of peripheral hormone effects with a beta-blocker, glucocorticoids to inhibit peripheral T4-to-T3 conversion and cover possible relative adrenal insufficiency, and aggressive supportive care with control of precipitants [3,11]. Propylthiouracil was chosen in the acute phase for its additional inhibition of peripheral conversion, with subsequent transition to methimazole for maintenance, in line with current guidelines [3]. One nuance merits reflection: amiodarone (300 mg) was administered during resuscitation, before the thyrotoxic etiology was apparent. Amiodarone carries a substantial iodine load and is itself a recognized cause of thyrotoxicosis and thyroid storm [11]; its use in the peri-arrest setting was unavoidable, but this underscores the value of early thyroid function testing in unexplained arrest, both to identify a reversible cause and to inform subsequent antiarrhythmic choices.

Finally, the decision to implant an S-ICD warrants discussion. The 2022 European Society of Cardiology guidelines state that the need for prophylactic defibrillator implantation in survivors of cardiac arrest attributed to a reversible cause should be considered according to the underlying cardiac disease and an individual evaluation of the future risk of life-threatening ventricular arrhythmia [16], and in the sections we reviewed, they do not specifically address ventricular arrhythmia associated with thyroid storm or thyrotoxicosis. The decision in this case therefore represents an individualized risk-benefit assessment rather than an established guideline-based recommendation. In thyrotoxicosis, reversibility is neither immediate nor guaranteed: antithyroid drug titration takes weeks, and recurrence of ventricular fibrillation has been documented in Graves' disease despite ongoing antithyroid therapy, including a recent case of poorly controlled, long-standing Graves' disease in which ventricular fibrillation recurred approximately two months after the index arrest and was terminated by an implanted transvenous defibrillator [17]. Higher free thyroxine has also been associated epidemiologically with sudden cardiac death [12]. These data are limited and do not allow us to state that a persistent arrhythmogenic substrate was present.

In our patient, the defibrillator was implanted approximately two months after the index event, before the ajmaline test, and the decision was grounded in the severity of the index event (ventricular fibrillation arrest as the first manifestation of the disease), persistent biochemical thyrotoxicosis at the time of the decision (free T3 above the reference range at months 1 and 2), uncertainty about the reversibility of the arrhythmogenic mechanism, and the absence of another identified trigger. Persistent TSH suppression alone does not indicate persistent arrhythmic risk, since TSH may remain suppressed after free hormone levels improve, and we did not rely on it. Definitive treatment by thyroidectomy is still awaited. We acknowledge that, if thyrotoxicosis was the sole cause, the arrest could be regarded as having a reversible cause, in which case defibrillator implantation might not have been required; this uncertainty is a limitation of our decision. As the patient was young, had no anticipated need for bradycardia or antitachycardia pacing, and faced decades of device exposure, the subcutaneous system was favored over a transvenous device to minimize lifetime lead-related and endovascular complications.

This report describes a single patient. Findings directly supported by the case include the following: ventricular fibrillation arrest; biochemical thyrotoxicosis with positive TSH-receptor antibodies and sonographic features of Graves' disease; absence of obstructive coronary disease, structural heart disease, and myocarditis; and a negative ajmaline provocation test. Findings that remain inferential include the following: the diagnosis of thyroid storm (BWPS 45 points with, and 15 points without, the post-arrest neurological component), the causal role of thyroid hormone excess in the arrhythmia, a possible contribution of coronary vasospasm that was not ruled out, the interpretation of the elevated transaminases, and the absence of Brugada syndrome, which a single negative provocation test cannot exclude. Pre-arrest laboratory data were not available, and the heart rate used for scoring was recorded after resuscitation, including epinephrine administration.

## Conclusions

Ventricular fibrillation arrest was the first manifestation of previously undiagnosed Graves' disease in this patient. This case highlights the importance of considering thyrotoxicosis in the etiological workup of unexplained cardiac arrest, as thyroid function testing is simple and may identify a potentially treatable cause. When thyroid hormone excess is the most plausible but unproven trigger, and a clearly established and reversible cause of the arrhythmia cannot be confirmed, management, including the decision on defibrillator implantation, should be guided by an individualized risk-benefit assessment.
